# Supplementary material for: Dual Impact of a Benzimidazole Resistant β-Tubulin on Microtubule Behavior in Fission Yeast
Source: Cells. 2021 Apr 28;10(5):1042. doi: 10.3390/cells10051042 (PMC8145593; doi:10.3390/cells10051042)
Supplement: Supplementary file 1 [file cells-10-01042-s001.zip › cells-1195473-supplementary.pdf]

Supplementary Information

Dual impact of a benzimidazole resistant β-tubulin on microtubule behavior in fission yeast

Mamika Minagawa, Minamo Shirato, Mika Toya and Masamitsu Sato

Supplementary table S1
Supplementary figures S1 and S2

Supplementary Table S1. *S. pombe* strains used in this study

Strains	Genotypes	Figures
MJ1043	*h90 nda3-TB101 leu1-32 ura4-D18 ade6-M216*	1A, 4A
KK0274	*h90 nda3-TB101-bsd leu1-32 ura4-D18 ade6-M216*	1A, 7A
KK0275	*h90 nda3-TB101-bsd leu1-32 ura4-D18 ade6-M216*	1A
KK0285	*h90 nda3-bsd leu1-32 ura4-D18 ade6-M216*	1A
KK0286	*h90 nda3-bsd leu1-32 ura4-D18 ade6-M216*	1A
JY0878	*h90 leu1-32 ura4-D18 ade6-M216*	1A, 4A, 7A
KK0174	*h90 Z2-GFP-atb2-kan leu1-32 ura4-D18 ade6-M216*	3A-B, 5A, 6A-C, 7B, 7D, 8C, S2C
KK0337	*h90 nda3-TB101-bsd Z2-GFP-atb2-kan leu1-32 ura4-D18 ade6-M216*	3A-B, 5A-B, 6A-C, 7B, 7D
SAK836	*h90 ade6-M216 leu1-32 ura4-D18 caf5::bsd pap1-del pmd1-del mfs1-del bfr1-del dnf2-del erg5::ura4*$^{+}$	4A
MN0136	*h90 nda3-TB101 ade6-M216 leu1-32 ura4-D18 caf5::bsd pap1-del pmd1-del mfs1-del bfr1-del dnf2-del erg5::ura4*$^{+}$	4A
MM0090	*h90 nda3-TB101-bsd Z2-GFP-atb2-kan leu1-32 ura4-D18 ade6-M216*	4B, S2C
YS0021	*h*$^{-}$ *ade6-M216 leu1-32 ura4-D18 caf5::bsd pap1-del pmd1-del mfs1-del bfr1-del dnf2-del erg5::ura4*$^{+}$ *Z2-GFP-atb2-kan*	4B, 5A
MN0129	*h*$^{-}$ *nda3-TB101 ade6-M216 leu1-32 ura4-D18 caf5::bsd pap1-del pmd1-del mfs1-del bfr1-del dnf2-del erg5::ura4*$^{+}$ *Z2-GFP-atb2-kan*	4B, 5A-C
KK0306	*h90 alp14::ura4*$^{+}$ *leu1-32 ura4-D18 ade6-M216*	7A
KK0335	*h90 nda3-TB101-bsd alp14::ura4*$^{+}$ *leu1-32 ura4-D18 ade6-M216*	7A
KK0173	*h90 alp14::ura4*$^{+}$ *Z2-GFP-atb2-kan leu1-32 ura4-D18 ade6-M216*	7B-D
KK0333	*h90 nda3-TB101-bsd alp14::ura4*$^{+}$ *Z2-GFP-atb2-kan leu1-32 ura4-D18 ade6-M216*	7B-D
KK0392	*h90 klp6::ura4*$^{+}$ *Z2-GFP-atb2-kan leu1-32 ura4-D18 ade6-M216*	8A, 8C
MN0051	*h90 nda3-TB101-bsd klp6::ura4*$^{+}$ *Z2-GFP-atb2-kan leu1-32 ura4-D18 ade6-M216*	8A, 8C
MJ0009	*h*$^{+}$ *leu1-32 ura4-D18 his2 ade6-M216*	8B, S2D
MJ0073	*h90 klp6::ura4*$^{+}$ *leu1-32 ura4-D18 ade6-M216*	8B
MN0214	*h*$^{-}$ *nda3-TB101 leu1-32 ura4-D18 ade6-M216*	8B

KK0386	*h90 nda3-TB101-bsd klp6::ura4⁺ leu1-32 ura4-D18 ade6-M216*	8B
KK0088	*h90 mal3-GFP-kan Z2-mCherry-atb2-hph leu1-32 ura4-D18 ade6-M216*	S2A
KK0317	*h90 nda3-TB101-bsd mal3-GFP-kan Z2-mCherry-atb2-hph leu1-32 ura4-D18 ade6-M216*	S2A
MM0282	*h90 alp7-3GFP-kan Z2-mCherry-atb2-hph leu1-32 ura4-D18 ade6-M216*	S2B
MM0283	*h90 nda3-TB101-bsd alp7-3GFP-kan Z2-mCherry-atb2-hph leu1-32 ura4-D18 ade6-M216*	S2B
MM0100	*h90 mal3::ura4⁺ Z2-GFP-atb2-kan leu1-32 ura4-D18 ade6-M216*	S2C
MM0093	*h90 mal3::ura4⁺ nda3-TB101-bsd Z2-GFP-atb2-kan leu1-32 ura4-D18 ade6-M216*	S2C
MM0095	*h90 nda3-TB101-bsd leu1-32 ura4-D18 ade6-M216*	S2D
MM0098	*h⁻ mal3::ura4⁺ leu1-32 ura4-D18 ade6-M216*	S2D
MM0099	*h⁻ mal3::ura4⁺ nda3-TB101-bsd leu1-32 ura4-D18 ade6-M216*	S2D

Origin of strains: the original *nda3-TB101* strain is a gift from M. Yamamoto [1]. SAK836 is a gift from S. Kawashima [2]. JY878 is our stock. Other strains were generated in this study.

Supplementary figures

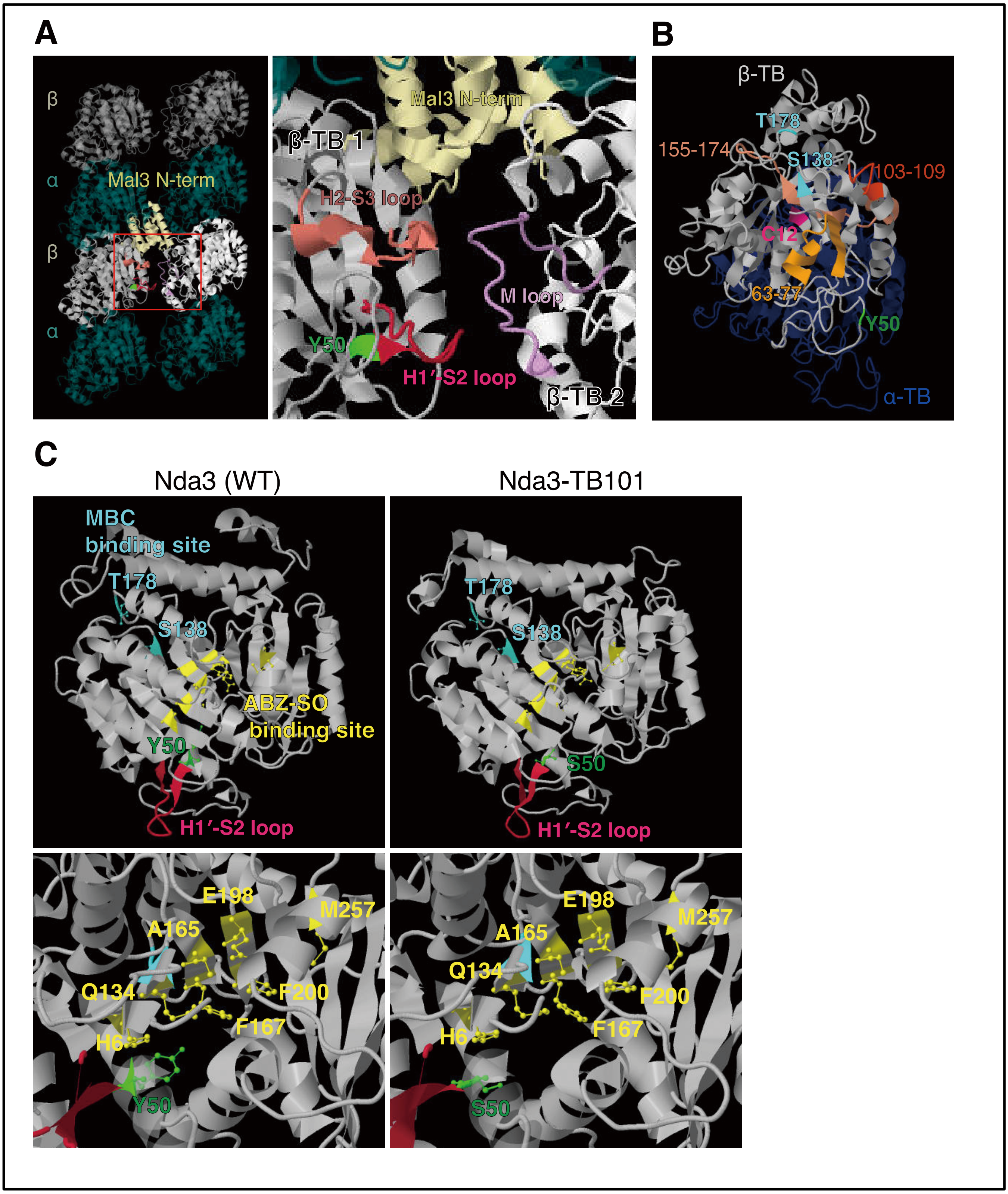

Supplementary figure 1. Relationship of Y50 and other major residues

(A) Alignment of mammalian αβ dimers in the B-type lattice in a back view from the putative inside of the microtubule. Emerald, α-tubulin (α-TB); white, β-tubulin (β-TB 1 and 2), based on the model proposed in previous study [3]. N-terminal fragment of Mal3 (ivory) binds to the corner of a tetramer from outside of the microtubule. Inset (red) is magnified in the right. Representative loops are highlighted in colors: H1′-S2 loop (red), H2-S3 loop (coral) and M loop (purple). Y50 (green) is located close to the H1′-S2 loop.

(B) Regions for GTP-binding in β-tubulin, as summarized in [4,5]. Indigo, α-tubulin (α-TB); gray, β-tubulin (β-TB). C12 (magenta) and 63–77 (orange) are located by the guanine base of GTP, the region, 155–174 (coral) is by the ribose. 103–109 (red) is thought to affect the GTP hydrolysis. S138 and T178 (turquoise) are putative MBC binding sites that might affect GTP binding, according to discussion in the previous study [6].

(C) Geographical relationship between Y50 and other candidates of benzimidazole binding sites shown with ball-and-stick modeling. S138 and T178 (turquoise) are as in B. Residues H6, Q134, A165, F167, E198, F200 and M257 (yellow) and Y50 (green) are putative binding site of ABZ-SO [7]. left, Nda3 WT (green, Y50); right, Nda3-TB101 (green, S50).

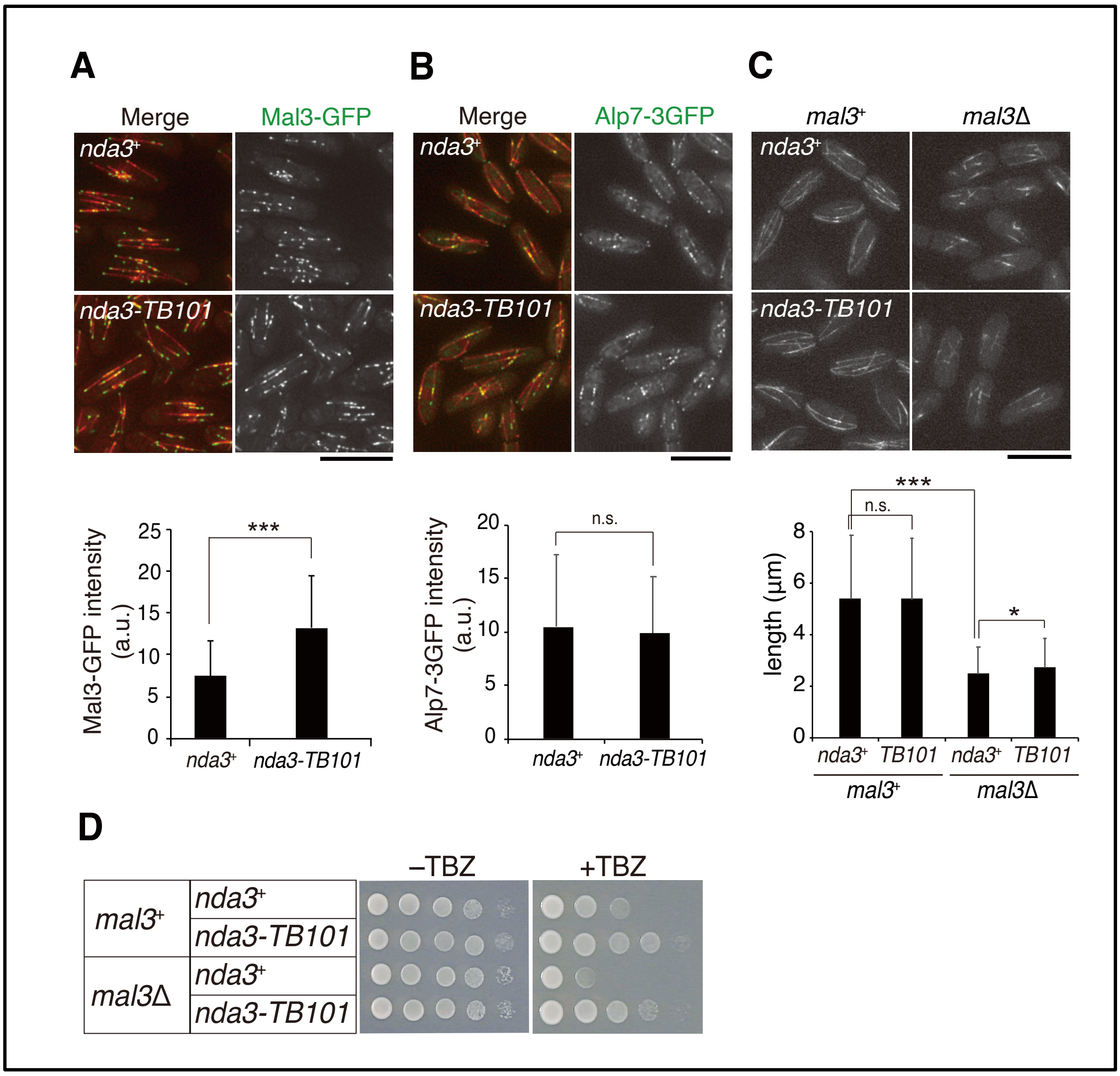

Supplementary figure 2. A structural model for αβ-tubulin in MT and positions of associating Mal3 and GTP

(A) Localization of Mal3-GFP (green) to cytoplasmic microtubules (mCherry-Atb2, red) in WT and *nda3-TB101* cells. Fluorescence intensity of Mal3-GFP at plus-end of microtubules were quantified. *nda3*$^+$ (WT), n = 40 tip signals; *nda3-TB101*, n = 39. ***p = 7.3E–06 < 0.001 (Welch's t-test); error bars, S.D.

(B) Localization of Alp7-3GFP (green) observed and quantified as in A. *nda3*$^+$ (WT), n = 40 tip signals; *nda3-TB101*, n = 42. n.s., not significant (Student's t-test); error bars, S.D.

(C) Organization of cytoplasmic microtubules in *mal3*Δ *nda3-TB101* cells. Cytoplasmic microtubules were short in *mal3*Δ cells as shown previously [8]. This was almost similar in *mal3*Δ *nda3-TB101* cells. *mal3*$^+$ *nda3*$^+$, n = 197 bundles; *mal3*$^+$ *nda3-TB101*, n = 198; *mal3*Δ *nda3*$^+$, n = 166; *mal3*Δ *nda3-TB101*, n = 163. n.s. not significant; *p = 0.036 < 0.05 (Student's t-test); ***p = 3.4E–38 < 0.001(Welch's t-test); error bars, S.D. Scale bars, 10 μm.

(D) Assays for TBZ sensitivity. The *mal3*Δ strain was sensitive to TBZ, but the *mal3*Δ *nda3-TB101* strain was resistant to TBZ. Ten-fold serial dilutions of indicated strains were spotted onto YE5S plates without and with 15 μg/ml TBZ for 3 days.

References

1. Yamamoto, M. Genetic analysis of resistant mutants to antimitotic benzimidazole compounds in *Schizosaccharomyces pombe*. *Mol Gen Genet* **1980**, *180*, 231–234.
2. Aoi, Y.; Sato, M.; Sutani, T.; Shirahige, K.; Kapoor, T. M.; Kawashima, S. A. Dissecting the first and the second meiotic divisions using a marker-less drug-hypersensitive fission yeast. *Cell Cycle* **2014**, *13*, 1327–1334.
3. Maurer, S. P.; Fourniol, F. J.; Bohner, G.; Moores, C. A.; Surrey, T. EBs recognize a nucleotide-dependent structural cap at growing microtubule ends. *Cell* **2012**, *149*, 371–382.
4. Nogales, E.; Wolf, S. G.; Downing, K. H. Structure of the αβ tubulin dimer by electron crystallography. *Nature* **1998**, *391*, 199–203.
5. Linse, K.; Mandelkow, E. M. The GTP-binding peptide of β-tubulin. Localization by direct photoaffinity labeling and comparison with nucleotide-binding proteins. *J Biol Chem* **1988**, *263*, 15205–15210.
6. Vela-Corcía, D.; Romero, D.; de Vicente, A.; Pérez-García, A. Analysis of β-tubulin-carbendazim interaction reveals that binding site for MBC fungicides does not include residues involved in fungicide resistance. *Sci Rep* **2018**, *8*, 7161.
7. Robinson, M. W.; McFerran, N.; Trudgett, A.; Hoey, L.; Fairweather, I. A possible model of benzimidazole binding to β-tubulin disclosed by invoking an inter-domain movement. *J Mol Graph Model* **2004**, *23*, 275–284.
8. Beinhauer, J. D.; Hagan, I. M.; Hegemann, J. H.; Fleig, U. Mal3, the fission yeast homologue of the human APC-interacting protein EB-1 is required for microtubule integrity and the maintenance of cell form. *J Cell Biol* **1997**, *139*, 717–728.
